# Epigenomic Characterization of Fibroblasts in Darier Disease Identifies Cross-cell-type Regulatory Gene Signatures

**DOI:** 10.2340/actadv.v106.adv-2026-0626

**Published:** 2026-10-28

**Authors:** Nuoqi Wang, Matthew Hunt, Monica Torres, Etty Bachar-Wikstrom, Jakob D. Wikstrom

**Affiliations:** 1 Dermatology and Venereology Division, Department of Medicine (Solna), Karolinska Institutet, Stockholm, Sweden; 2 Dermato-Venereology Clinic, Karolinska University Hospital, Stockholm, Sweden

**Keywords:** methylation, RNA-seq, Darier disease, skin

## Abstract

Darier disease is a monogenic acantholytic dermatosis caused by mutations in *ATP2A2* that primarily disrupt keratinocyte calcium homeostasis. Whether dermal fibroblasts harbour disease-associated epigenetic alterations remains uncharacterized. In this study, we performed genome-wide DNA methylation profiling on primary fibroblasts from lesional and nonlesional skin, followed by STRING protein–protein interaction network analysis to identify regulatory hub genes. Hub gene expression was validated by qPCR and cross-referenced with single-cell RNA-seq data from healthy skin and published RNA-seq data from Darier disease keratinocytes. Lesional fibroblasts showed consistent, site-specific hyper- and hypomethylation converging on inter-connected regulatory hubs. Hypomethylated hub genes included transcription factors and signalling integrators while hypermethylated hub genes comprised growth-regulatory and adhesion-related genes. Methylation direction predicted transcriptional output at 10 of 12 hub genes by qPCR. These hub genes are expressed across both fibroblast and keratinocyte populations in healthy skin, and lesional keratinocyte RNA-seq data showed directionally concordant expression changes, indicating shared transcriptional dysregulation across compartments. These findings suggest that Darier disease involves a stable fibroblast epigenetic state with cross-cell-type transcriptional relevance, potentially contributing to dermal–epidermal regulatory disruption in lesional skin.

**SIGNIFICANCE**

Darier disease is a rare inherited skin condition that causes painful, recurring sores that commonly reappear in the same locations. But no one fully understands why. We studied skin cells that support the skin from beneath, and found that in affected areas, these cells carry stable chemical marks on their DNA that change how genes behave. Remarkably, the same genes are also altered in the surface skin cells. This suggests that Darier disease affects more than one type of skin cell. These chemical marks may help explain why sores keep coming back, potentially opening new directions for treatment.

Darier disease (DD) is a rare autosomal dominant genodermatosis characterized by keratotic papules and plaques, with an estimated prevalence ranging from 1 in 100,000 to 1 in 30,000 individuals worldwide (1; 2). DD is caused by mutations in the *ATP2A2* gene located on chromosome 12q23-24.1 (3) which encodes the sarco/endoplasmic reticulum calcium ATPase type 2 isoform (SERCA2) (3). As a critical pump responsible for transporting cytosolic Ca²^+^ into the endoplasmic reticulum, SERCA2 plays an indispensable role in maintaining intracellular calcium (Ca^2+^) homeostasis (4). Disruption of this delicate Ca²^+^ balance is considered the primary mechanism of DD (5), leading to impaired differentiation of keratinocytes and compromised desmosomal integrity (6), which ultimately results in the characteristic histological hallmarks of acantholysis and dyskeratosis (7). Clinically, this manifests as greasy, hyperkeratotic papules predominantly distributed in seborrheic areas, often accompanied by nail abnormalities and mucous membrane involvement (8).

DNA methylation is one of the most extensively studied epigenetic modifications, and plays a fundamental role in regulating gene expression without altering the underlying DNA sequence. By adding methyl groups to cytosine residues within CpG dinucleotides, this modification can influence chromatin structure and transcriptional activity, thereby modulating critical cellular processes such as differentiation, proliferation, and apoptosis (9, 10). In the context of skin biology, DNA methylation has been implicated as a key regulator of epidermal development, keratinocyte differentiation (11), and skin barrier formation (12). Precise control of methylation patterns is essential for the functional epidermal barrier (13). Accumulating evidence has demonstrated that aberrant DNA methylation patterns are associated with the pathogenesis of various dermatological conditions (14). Genome-wide methylation studies have revealed distinct epigenetic signatures in psoriasis (15), where differentially methylated regions are enriched in genes involved in keratinocyte proliferation and immune responses. Similarly, altered methylation profiles have been documented in atopic dermatitis (16) and cutaneous squamous cell carcinoma (17). These findings underscore the critical role of epigenetics in skin homeostasis and disease.

However, despite the well-established genetic basis of DD and the recognized importance of epigenetic regulation in skin pathophysiology, genome-wide DNA methylation profiling in DD remains unexplored. To date, few studies have systematically investigated whether epigenetic alterations contribute to the observed phenotypic variability of *ATP2A2* mutations. Given the central role of Ca²^+^ signalling in both differentiation and epigenetic regulation, it is plausible that DNA methylation changes may intersect with the primary genetic defect.

A particularly compelling clinical hallmark of Darier disease is the marked tendency of lesions to recur at the same anatomical sites (5, 18). This characteristic implies that local skin cells may retain a persistent, site-specific biological memory. This observation raises the possibility that stable epigenetic marks rather than transient inflammatory or microenvironmental signals underlie the spatial patterning of DD disease manifestation. Fibroblasts, as long-lived stromal cells that persist within the dermal compartment independently of epidermal dynamics, represent an ideal model to interrogate such cell-intrinsic memory. By examining primary fibroblasts in culture, after removal from the *in vivo* inflammatory and epidermal microenvironment, we could specifically determine whether stable epigenetic differences are maintained within the dermal compartment itself.

Here, we present genome-wide DNA methylation profiling of fibroblasts from DD patients and healthy controls. We constructed protein–protein interaction (PPI) networks for hypermethylated and hypomethylated genes, evaluated promoter and gene body methylation patterns, and identified hub genes through network analysis. Candidate genes were validated by qPCR and cross-validated with RNA-seq data from keratinocyte-rich biopsies of the same patients (19). This integrated approach aims to uncover epigenetic modifiers contributing to DD pathogenesis and phenotypic heterogeneity.

## MATERIALS AND METHODS

### Patient samples and primary fibroblast culture

Full-thickness skin punch biopsies were obtained from lesional (L) and adjacent nonlesional (NL) skin of 12 DD patients. Primary DD dermal fibroblasts (DDF) were isolated by explant outgrowth. Briefly, dermal tissue was separated from epidermis, minced into small fragments, and cultured in DMEM glucose-free media (Thermo Fisher Scientific), supplemented with 10% fetal bovine serum (Thermo Fisher Scientific) and antibiotics (100 units/ml penicillin and 100 µg/ml streptomycin) (both Thermo Fisher Scientific) at 37°C with 5% CO₂. DDF migrating from explants were expanded and passaged using standard trypsinization. Cells between passages 3–6 were used for all experiments.

### RT-qPCR

Total RNA was extracted from cells using Trizol reagent (Thermo Fisher Scientific). After determining the sample quality, reverse transcription was performed using the RevertAid First Strand cDNA Synthesis Kit (Thermo Fisher Scientific). Gene expression was quantified by SYBR Green expression assays and normalized to *β-actin*. The primer information used in these experiments is listed in Table SI .

### Genome-wide DNA methylation profiling

Genomic DNA was extracted from cultured DDF using the QIAquick Gel Extraction Kit (QIAGEN), according to the manufacturer’s protocol. DNA concentration and purity were assessed by spectrophotometry.

Genome-wide DNA methylation profiling was performed using the Illumina Human Methylation EPIC, following standard protocols. Raw intensity data were processed in R (4.4.3) using the minfi package (1.54.1). Probes with detection *p*>0.01, cross-reactive probes, probes containing SNPs, and sex chromosome probes were removed. Data were normalized using BMIQ.

Differentially methylated CpG sites between L and NL DDF were identified using paired linear modelling with the limma package. CpG sites with adjusted *p*<0.05 and β difference ≥0.10 were considered significant.

### Genomic annotation and enrichment analysis

DMPs were annotated to genomic features including promoter regions (TSS200, TSS1500, 1stExon, 5’UTR), gene bodies and untranslated regions using Illumina annotation files. Enrichment of hypermethylated and hypomethylated regions across genomic categories was assessed by odds ratios relative to the genomic background with 95% confidence intervals.

### STRING network construction

Genes associated with significantly hypermethylated and hypomethylated CpG sites were analysed separately for protein–protein interaction (PPI) network construction. Protein interaction data were retrieved from the STRING database using the STRINGdb R package (v11.5). Gene symbols were mapped to STRING identifiers using the map function, and interaction networks were extracted with a minimum confidence score of 0.5 (STRING score≥500).

### Healthy skin single-cell RNA-seq analysis

Publicly available single-cell RNA sequencing data from full-thickness healthy human skin biopsies (dataset accession GSM8779712) were analysed using Seurat v5. Cells were filtered based on mitochondrial gene percentage and gene counts, normalized and integrated across donors using Harmony.

Cell clusters were annotated according to canonical markers to identify keratinocyte and fibroblast populations. Expression of methylation-prioritized genes was visualized using feature plots and summarized across cell types.

## RESULTS

### Comprehensive overview of DNA methylation profiling in Darier disease fibroblasts

To characterize the global DNA methylation landscape in DD, we performed genome-wide methylation analysis on primary fibroblasts derived from L and NL skin of DD patients (Fig. 1A). Initial quality control assessments confirmed the robustness of the dataset. Briefly, all samples exhibited high hybridization efficiency and signal-to-noise ratios (Fig. S1A,B), successful and uniform bisulphite conversion (Fig. S1C), and effective normalization eliminating probe-type bias (Fig. S1D,E). Principal component analysis revealed no significant outliers, and the primary variation within the dataset appeared to correspond to the lesion status, with no overt confounding batch effects detected (Fig. S1F).

**Fig. 1.**
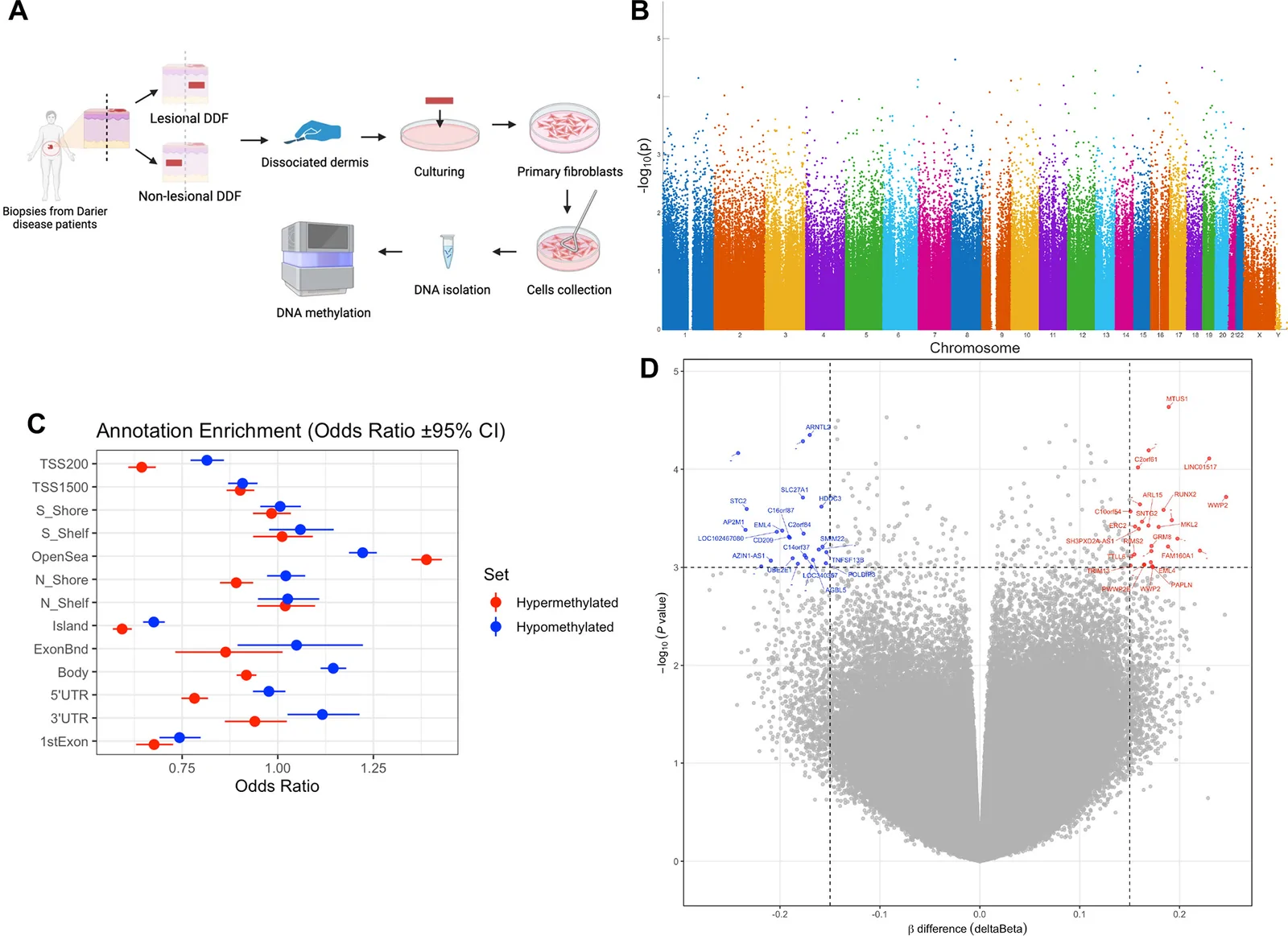
Genome-wide DNA methylation landscape of fibroblasts from Darier disease lesional and nonlesional skin. (**A**) Schematic summary of the experimental setup. (**B**) Manhattan plot displaying genome-wide distribution of CpG sites analysed in lesional vs nonlesional DDF. (**C**) Distribution of differentially methylated CpG sites (DMPs) across genomic features. (**D**) Volcano plot illustrating differential DNA methylation between lesional and nonlesional DDF in a paired analysis.

An unbiased overview of the differentially methylated positions (DMPs) is presented in **Fig. 1**. The Manhattan plot (Fig. 1B) illustrates the genome-wide distribution of CpG sites demonstrating altered methylation. The volcano plot (Fig. 1D) summarizes the magnitude (β difference) and statistical significance of these changes, distinguishing sites with more subtle (β difference <0.15) and pronounced (β difference >0.15) differential methylation. To characterize the genomic context of DNA methylation changes in DDF, we performed annotation enrichment analysis of differentially methylated regions (DMRs) identified between L and NL primary DDF. Hypermethylated and hypomethylated DMRs were analysed separately, and enrichment was assessed using odds ratios relative to the genomic background (Fig. 1C). Overall, hypermethylated and hypomethylated DMRs exhibited largely similar genomic distributions, with odds ratios close to unity across most annotation categories. Despite the broadly overlapping patterns, subtle directional tendencies were observed. Hypomethylated DMRs showed a tendency toward enrichment in promoter-associated regions, including TSS200, TSS1500 and CpG islands, whereas hypermethylated DMRs displayed a relative shift toward distal genomic regions such as open sea regions and gene bodies. These findings indicate that DNA methylation alterations in L DDF from DD patients are widespread but moderate in magnitude, without pronounced segregation between hypermethylated and hypomethylated regions. A focused examination of genes associated with the most significantly hyper- and hypomethylated CpG sites is provided in Tables SII and SIII. The top 10 genes in each category were annotated for their known biological functions. This preliminary functional association analysis suggests that the observed methylation alterations could potentially influence pathways relevant to cell adhesion, Ca^2+^ signalling, and keratinocyte differentiation.

### Network-based characterization of differentially methylated genes

To further explore the potential functional organization of differentially methylated CpG sites in lesional fibroblasts, we mapped genes associated with significantly hypermethylated and hypomethylated CpGs onto PPI networks using the STRING database. The hypomethylated gene network formed a densely connected PPI structure, with multiple nodes showing high degrees of connectivity (**Fig. 2**A). In contrast, the hypermethylated gene network was comparatively smaller and more fragmented, consisting of several discrete interaction modules (Fig. 2B).

**Fig. 2.**
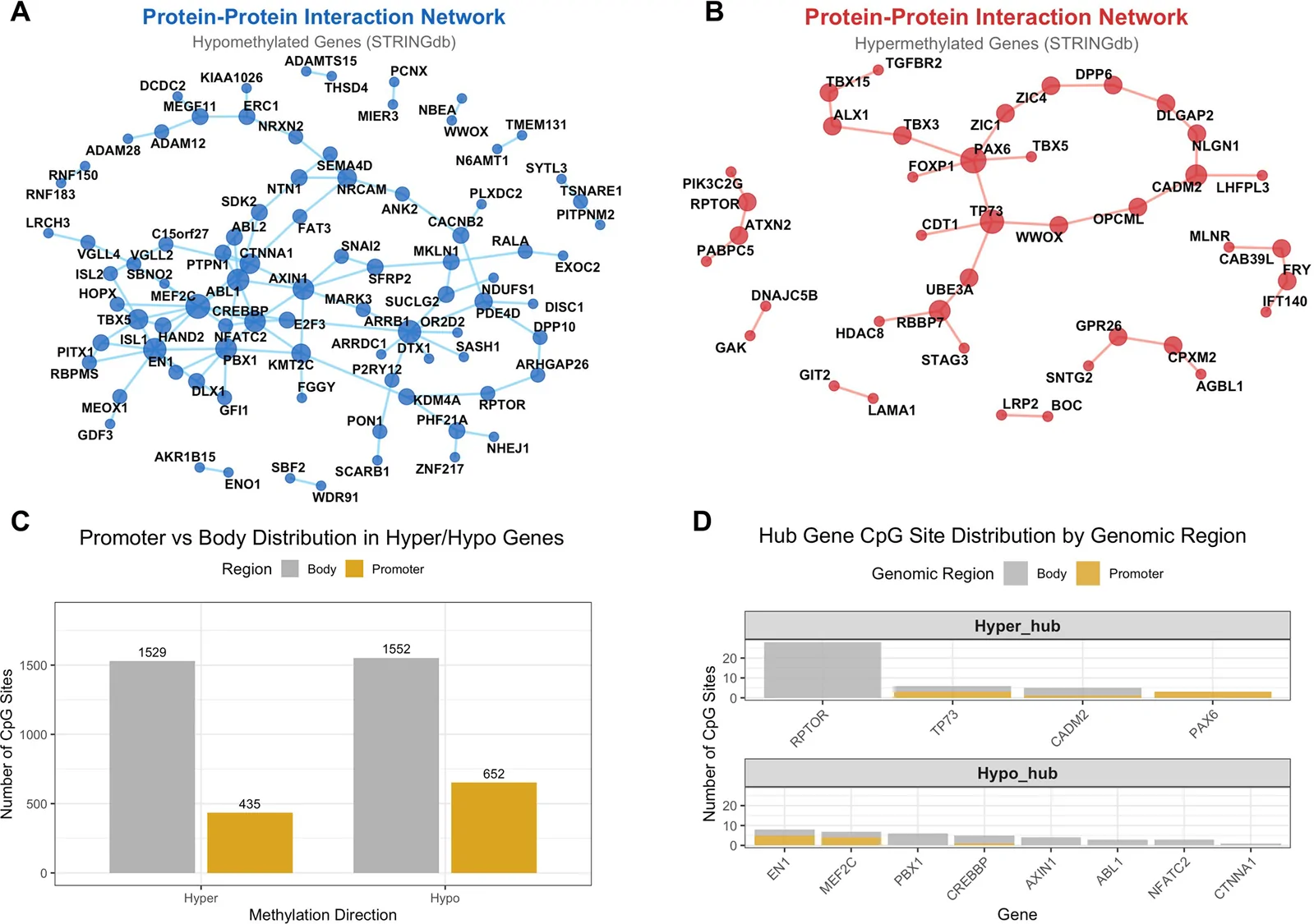
Functional characterization of significantly differentially methylated CpG sites in lesional DDF. (**A**) STRING PPI network constructed from genes associated with hypomethylated CpG sites. Nodes represent genes and edges indicate known or predicted protein–protein interactions. (**B**) STRING PPI network constructed from genes associated with hypermethylated CpG sites. (**C**) Distribution of CpG sites across promoter regions and gene bodies for all genes represented in the STRING networks, shown separately for hypomethylated and hypermethylated gene sets. (**D**) Gene-level distribution of CpG sites across promoter regions and gene bodies for highly connected hub genes identified within the STRING networks. Individual bars represent hub genes, grouped by methylation direction.

Having established distinct PPI networks for hypermethylated and hypomethylated genes, we next examined whether CpG sites associated with these networks preferentially localized to promoter regions or gene bodies (Fig. 2C). While CpG sites associated with all network genes were predominantly located within gene bodies, hypomethylated network genes exhibited a relatively higher representation of promoter-associated CpG sites compared with hypermethylated network genes.

Because regulatory effects are more likely to be mediated by highly connected genes, we next focused on hub genes within the PPI networks (Fig. 2D). Hypomethylated hub genes were enriched for transcriptional regulators and signalling-related genes, including transcription factors (*MEF2C*, *PBX1*, *EN1*, *NFATC2*), transcriptional coactivators (*CREBBP*), and signalling components involved in Wnt and cytoskeletal regulation (*AXIN1*, *ABL1*), as well as cell–cell adhesion molecules (*CTNNA1*). In contrast, hypermethylated hub genes predominantly comprised genes involved in cellular growth regulation and developmental transcriptional control, including components of the mTOR signalling pathway (*RPTOR*), p53 family-associated transcription factors (*TP73*), developmental regulators (*PAX6*), and cell adhesion molecules (*CADM2*). For these genes, *EN1*, *MEF2C*, *TP73* and *PAX6* were mainly located within promoter-associated CpGs. Thus network-based analysis reveals DNA methylation changes in lesional fibroblasts are organizationally non-random. Hypomethylation concentrated at promoter-proximal CpGs of transcriptional activators and hypermethylation enriched in gene bodies of growth-regulatory and adhesion-related genes.

### Hub gene RNA expression

To assess whether methylation changes observed in network-identified genes were associated with altered transcriptional output, we detected mRNA expression in primary DDF derived from lesional and nonlesional DD skin (Fig. 3A).

**Fig. 3.**
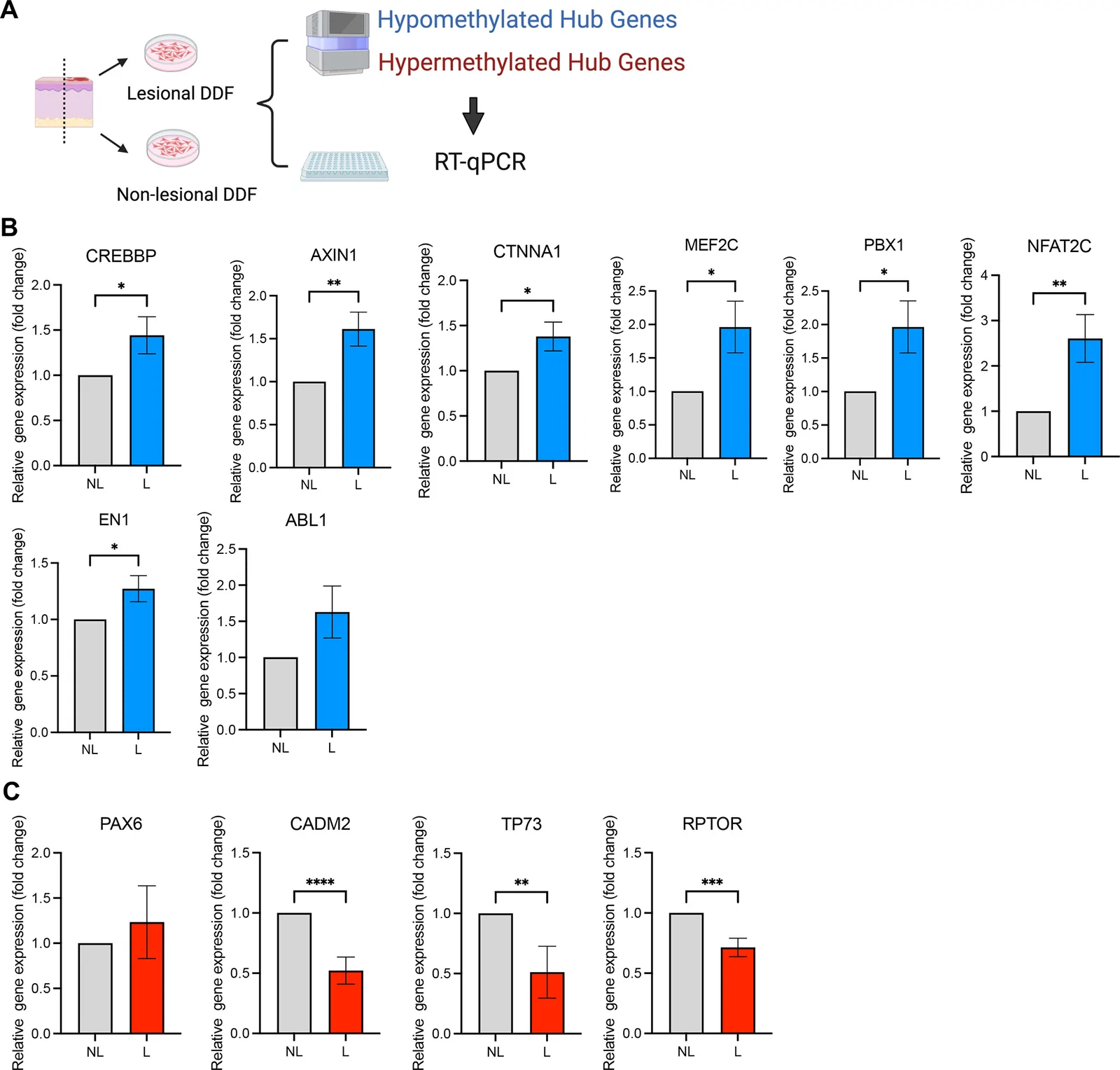
Relative mRNA expression of lesional and nonlesional DDF methylated hub genes. (**A**) Schematic representation of the experimental setup. (**B-C**) Quantification (mean±SEM) of relative gene expression of (**B**) hypomethylated and (C) hypermethylated hub genes in primary lesional (L) and nonlesional (NL) DDF. *N*=12 biological replicates containing 3 technical replicates. Statistical significance was determined by unpaired t test; *p*<0.05 (*), *p*<0.01 (**), *p*<0.001 (***), *p*<0.0001 (****).

Among the selected hypomethylated genes, *CREBBP*, *AXIN1*, *CTNNA1*, *MEF2C*, *PBX1*, *NFATC2* and *EN1* (**Fig. 3**A) showed significantly increased mRNA expression in L DDF compared with NL DDF. In contrast, hypermethylated genes *CADM2*, *TP73* and *RPTOR* (Fig. 3B) exhibited significantly reduced expression in lesional samples. No statistically significant differences were observed for *ABL1* or *PAX6* (Fig. 3C) between L and NL DDF. Notably, the direction of expression changes was consistent with the overall methylation direction (hypermethylation or hypomethylation) observed for these genes in L DDF. Thus, mRNA expression of 10 out of 12 network-identified hub genes was significantly altered in L DDF in a direction concordant with their methylation status, demonstrating that the observed epigenetic changes are functionally coupled to transcriptional output.

### Expression patterns of methylation-prioritized genes in healthy skin single-cell transcriptomes and DD keratinocytes

To determine whether the methylation-associated transcriptional changes identified in lesional fibroblasts are also present in keratinocytes, we analysed publicly available single-cell RNA sequencing data from full-thickness skin biopsies of 6 healthy individuals (GSM8779712). Cell clustering identified major epidermal and dermal populations, including keratinocytes and fibroblasts (**Fig. 4**A).

**Fig. 4.**
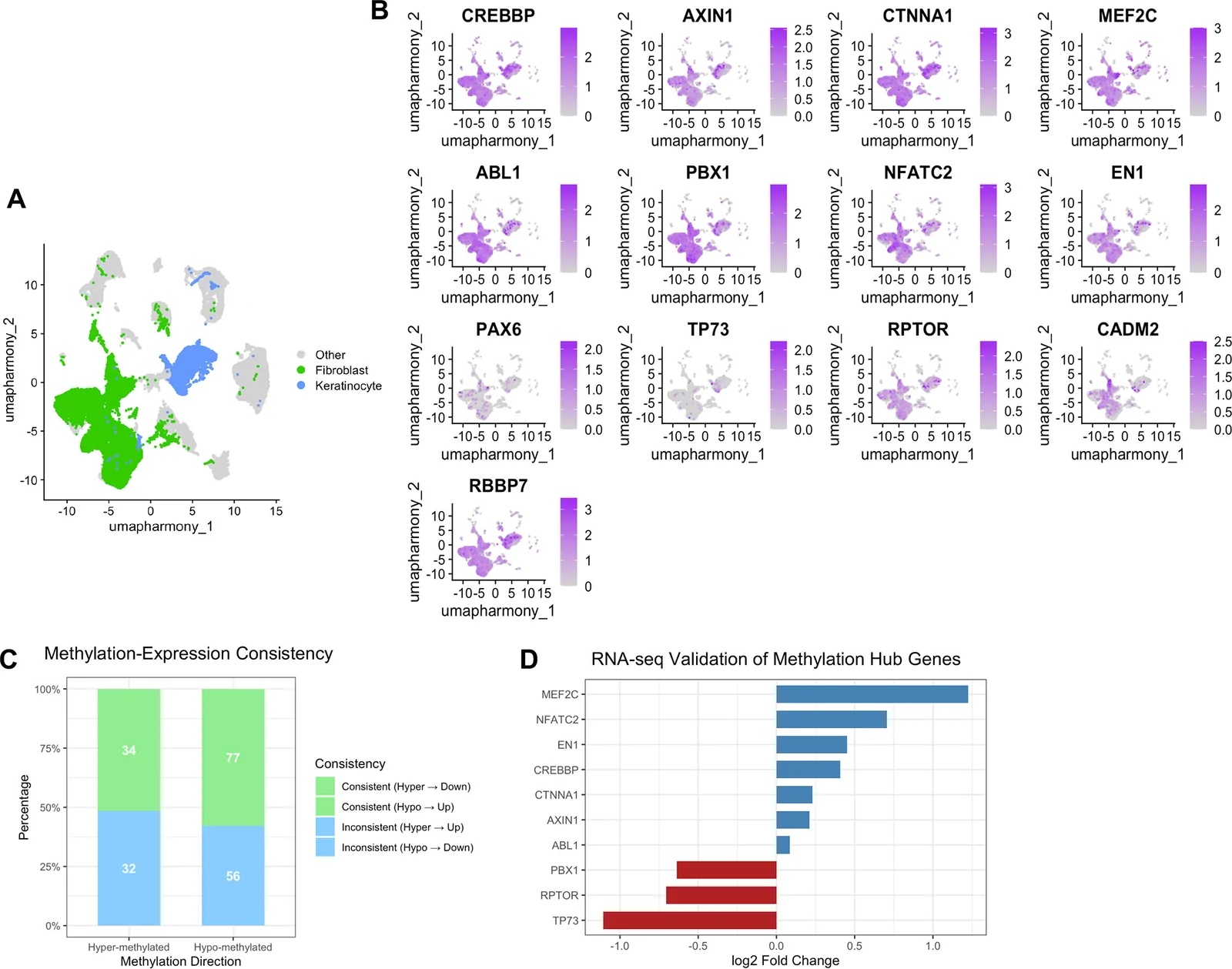
Integration of fibroblast methylation hubs with single-cell expression context and RNA-seq validation in Darier disease. (**A**) UMAP projection of healthy human skin single-cell RNA-seq data showing major cell populations, including fibroblasts and keratinocytes. Cells are coloured by annotated cell type. (**B**) Feature plots displaying normalized expression of methylation-prioritized hub genes across fibroblast and keratinocyte populations in healthy skin single-cell data. (**C**) Directional consistency between differential DNA methylation in cultured fibroblasts and gene expression changes. (**D**) RNA-seq validation of selected methylation hub genes in Darier disease patient biopsies (predominantly keratinocytes). Log2 fold changes between lesional and nonlesional tissue are shown.

Among genes showing increased expression in lesional fibroblasts by qPCR, *CREBBP*, *AXIN1*, *CTNNA1*, *MEF2C*, *NFATC2*, *ABL1* and *EN1* were expressed in both fibroblasts and keratinocytes, with variable enrichment across cell types. In contrast, *RPTOR* and *CADM2*, which were downregulated in lesional fibroblasts, exhibited lower or more restricted expression patterns, particularly within fibroblast populations. Similarly, *TP73* also displayed lower expression, with restricted patterns almost exclusively in keratinocyte clusters. *PAX6*, which showed no significant expression differences by qPCR, displayed low or heterogeneous expression in the single-cell dataset, consistent with limited transcriptional changes observed in bulk fibroblast analysis (Fig. 4B). Thus, the majority of hub genes showing methylation-associated transcriptional changes in lesional fibroblasts are broadly expressed across both fibroblast and keratinocyte populations in healthy skin, indicating a potential correlative link of their involvement in dermal–epidermal regulatory crosstalk.

To assess whether methylation-associated transcriptional changes identified in cultured L DDF are also detectable in patient keratinocytes, we examined RNA-seq expression of prioritized hub genes in Darier disease biopsies (predominantly keratinocytes) (19). Overall, methylation direction showed concordance with gene expression changes. Hypomethylated genes were more frequently upregulated (77/133), whereas approximately half of hypermethylated genes showed decreased expression (34/66), indicating modest but reproducible methylation–expression coupling (Fig. 4C). Thus, genome-wide methylation direction predicts transcriptional output at a rate significantly above chance in both hypomethylated (58%) and hypermethylated (52%) gene sets, confirming reproducible methylation-expression coupling across the fibroblast epigenome.

Consistent with fibroblast qPCR results, several hypomethylated hub genes, including *CREBBP*, *AXIN1*, *CTNNA1*, *MEF2C*, *NFATC2*, and *EN1* were upregulated in lesional keratinocytes, with *MEF2C* and *NFATC2* showing the strongest increases. Conversely, hypermethylated hubs such as *TP73*, *RPTOR*, and *PBX1* were downregulated. *ABL1* expression remained near baseline, consistent with the lack of change observed in fibroblasts (Fig. 4D). Thus, concordant expression changes at key hub genes indicating that these regulatory nodes are dysregulated across cell types in lesional DD skin.

Together, these findings both indicate that methylation-driven transcriptional changes in lesional fibroblasts are recapitulated in lesional keratinocytes, and that the affected hub genes: spanning Ca^2+^-responsive transcription (*NFATC2*, *MEF2C*), Wnt-mediated cell fate (*AXIN1*), mTOR-dependent homeostasis (*RPTOR*), and adhesion remodeling (*CTNNA1*, *CADM2*) may converge on pathological axes relevant to the Ca^2+^ dysregulation, impaired differentiation, and barrier instability characteristic of DD.

## DISCUSSION

This study provides the first genome-wide analysis of DNA methylation in primary fibroblasts derived from lesional vs nonlesional skin of DD patients. A central clinical feature of DD is the tendency of lesions to recur at the same anatomical sites, suggesting that local skin cells may retain a persistent, site-specific biological memory. By profiling primary DDF expanded *in vitro* after removal from the *in vivo* inflammatory and epidermal microenvironment, we specifically asked whether such memory is encoded at the epigenetic level within the dermal compartment. The retention of differential methylation following *in vitro* expansion strongly supports the presence of a durable, cell-intrinsic fibroblast epigenetic state distinguishes lesional from nonlesional skin independently of the local tissue environment.

### Methylation alterations converge on regulatory hub genes with distinct genomic architectures

Differentially methylated positions were not randomly distributed but converged on highly interconnected regulatory genes after STRING PPI network construction. The hypomethylated gene set formed a densely connected network, with 8 hub genes identified by degree centrality: transcription factors *MEF2C*, *PBX1* (20), *EN1* (21), and *NFATC2* (22); transcriptional coactivator *CREBBP (23*); Wnt pathway component *AXIN1*; cytoskeletal kinase *ABL1*; and adhesion molecule *CTNNA1*. The hypermethylated network was comparatively smaller and more fragmented, yielding 4 hubs: the mTOR pathway component *RPTOR*; p53-family transcription factor *TP73*; developmental regulator *PAX6*; and cell adhesion molecule *CADM2*.

The opposing methylation patterns at *CTNNA1* (hypomethylated) and *CADM2* (hypermethylated) are particularly informative. Both encode adhesion-related proteins, yet their divergent epigenetic states do not reflect contradictory effects on adhesion per se. Rather, *CTNNA1* links cadherin complexes to the actin cytoskeleton and participates in Wnt signal transduction (24), while *CADM2* mediates immunoglobulin-family-dependent intercellular contacts (25). This pattern reflects compositional remodeling of the adhesion program rather than a net gain or loss of adhesive function. Importantly, concordant transcriptional changes confirmed by qPCR in 7 of 8 hypomethylated hubs and 3 of 4 hypermethylated hubs establish that these methylation differences carry functional transcriptional weight, not merely statistical signal.

The identified hub genes do not converge on a single linear pathway but define a regulatory architecture spanning Wnt/β-catenin, Ca^2+^-calcineurin-NFAT, mTOR, and p53-family signalling. The concentration of methylation change at regulatory integrators with broad network connectivity rather than diffuse reprogramming across thousands of sites is consistent with the monogenic nature of DD. Unlike malignancies or complex developmental syndromes marked by widespread epigenetic disruption (26), DDF exhibit a focused shift in transcriptional wiring that likely reflects downstream adaptation to the primary *ATP2A2* defect rather than independent epigenetic instability.

### Fibroblasts as modulators of the lesional dermis

Although fibroblasts are not the primary genetically affected cell type in DD, they are central organizers of dermal–epidermal crosstalk. For example, *NFATC2* hypomethylation may represent a fibroblast-intrinsic adaptive response to the altered calcium microenvironment of lesional skin (27, 28). Similarly, increased *AXIN1* expression suggests an attenuation of Wnt signalling output in lesional fibroblasts, which may alter paracrine Wnt gradients that normally govern basal keratinocyte proliferation and epidermal homeostasis (29, 30). Upregulation of *EN1*, whose activation in fibroblasts promotes a pro-fibrotic, scar-forming state (31), raises the possibility that lesional fibroblasts adopt an altered matrix-remodelling phenotype contributing to lesion chronicity. Conversely, reduced *RPTOR* expression may impair fibroblast-mediated extracellular matrix synthesis and growth factor secretion and further damage epidermal integrity (32). These fibroblast-level changes are not isolated from the epidermal compartment. Cross-referencing with published lesional biopsy RNA-seq data revealed directionally concordant expression changes in 58% of hypomethylated and 52% of hypermethylated genes, with the strongest concordance at hub genes expressed in both fibroblasts and keratinocytes in healthy skin single-cell data. This cross-compartment alignment suggests that shared regulatory nodes operate across the dermal–epidermal axis in DD (33).

### Limitations

Several limitations require acknowledgment. The patient cohort was reasonably small (*N*=12 paired samples), reflecting the rarity of DD, and methylation differences were modest in magnitude. Biopsy-derived bulk RNA-seq limits cell-type-resolved interpretation. Future studies should incorporate single-cell and spatial epigenomic approaches to resolve compartment-specific regulatory states in lesional skin, and functional organotypic co-culture models will be essential to determine whether hub genes such as *EN1*, *NFATC2* and *AXIN1* causally modulate epidermal adhesion and lesion localization.

### Conclusion

Our findings support a model in which DD encompasses not only keratinocyte-intrinsic Ca^2+^ dysregulation but also stable, network-level modulation of dermal fibroblast regulatory identity. Lesional fibroblasts exhibit a refined shift in transcriptional control nodes that may shape the local tissue environment without global epigenomic instability. This dermal regulatory layer offers a conceptual expansion of DD pathobiology beyond a strictly epithelial disorder and identifies epigenetic hub genes as candidates for future mechanistic and therapeutic investigation.

## Data Availability

RNA-sequencing data is available in the following database: 582 https://www.ebi.ac.uk/biostudies/arrayexpress (Accession number: E-MTAB-13754). The data that support the findings of this study are available from the corresponding author upon reasonable request.

## Supplementary material

Figure S1.

Table SI.

## References

[1] Zagoras T , Inci R , Kantere D , Holmström P , Broström J , Gillstedt M , et al . Incidence and Prevalence of 73 Different Genodermatoses: A Nationwide Study in Sweden. Acta Derm Venereol 2023; 103: adv12404. 10.2340/actadv.v103.12404 PMC10464823 37615526

[2] Labbouz S , Dodiuk-Gad RP . Darier disease: Golden era of discovery and global collaborations. J Eur Acad Dermatol Venereol 2025; 39: 883–884. 10.1111/jdv.20641 PMC12023717 40277206

[3] Parfitt E , Burge S , Craddock N , Roberts E , McLean WH , Weissenbach J , et al . The gene for Darier’s disease maps between D12S78 and D12S79. Hum Mol Genet 1994; 3: 35–38. 10.1093/hmg/3.1.35 7512859

[4] Sánchez Martínez EM , Moneva Léniz LM , Gegúndez Hernández H , Mateu Puchades A . Darier Disease: A Case Series of 20 Patients and Review of the Literature. Actas Dermosifiliogr (Engl Ed) 2021: S0001-7310(21)00100-9. 10.1016/j.ad.2020.01.014 33675731

[5] Ettinger M , Kimeswenger S , Deli I , Traxler J , Altrichter S , Noack P , et al . Darier disease: Current insights and challenges in pathogenesis and management. J Eur Acad Dermatol Venereol 2025; 39: 942–951. 10.1111/jdv.20448 PMC12023721 39606894

[6] Celli A , Mackenzie DS , Zhai Y , Tu CL , Bikle DD , Holleran WM , et al . SERCA2-controlled Ca. J Invest Dermatol 2012; 132: 1188–1195. 10.1038/jid.2011.447 PMC3305850 22277942

[7] Bachar-Wikström E , Wikström JD . Darier Disease - A Multi-organ Condition? Acta Derm Venereol 2021; 101: adv00430. 10.2340/00015555-3770 PMC9364244 33606037

[8] Ashok Kumar P , Paulraj S , Dutta S . Debilitating Darier’s Disease and Its Impact on the Quality of Life. Cureus 2020; 12: e8133. 10.7759/cureus.8133 PMC7294863 32550053

[9] Dawson MA , Kouzarides T . Cancer epigenetics: from mechanism to therapy. Cell 2012; 150: 12–27. 10.1016/j.cell.2012.06.013 22770212

[10] Pop S , Enciu AM , Tarcomnicu I , Gille E , Tanase C . Phytochemicals in cancer prevention: modulating epigenetic alterations of DNA methylation. Phytochem Rev 2019; 18: 1005–1024. 10.1007/s11101-019-09627-x

[11] Botchkarev VA , Gdula MR , Mardaryev AN , Sharov AA , Fessing MY . Epigenetic regulation of gene expression in keratinocytes. J Invest Dermatol 2012; 132: 2505–2521. 10.1038/jid.2012.182 PMC3650472 22763788

[12] Rani A , Kaur G , Kumar R , Sachan RSK , Kumar M , Sable H , et al . Role of DNA Methylation in the Pathogenesis of Skin Disorders: Mechanisms, Inhibitors of Methylation-Related Enzyme, and Molecular Docking Studies. Biomed Res Int 2025; 2025: 7002918. 10.1155/bmri/7002918 PMC11968163 40182929

[13] Grönniger E , Max H , Lyko F . Skin Rejuvenation by Modulation of DNA Methylation. Exp Dermatol 2024; 33: e70005. 10.1111/exd.70005 39440959

[14] Dermitzakis I , Kyriakoudi SA , Chatzianagnosti S , Chatzi D , Vakirlis E , Meditskou S , et al . Epigenetics in Skin Homeostasis and Ageing. Epigenomes 2025; 9: 3. 10.3390/epigenomes9010003 PMC11755608 39846570

[15] Dopytalska K , Ciechanowicz P , Wiszniewski K , Szymańska E , Walecka I . The Role of Epigenetic Factors in Psoriasis. Int J Mol Sci 2021; 22: 9294. 10.3390/ijms22179294 PMC8431057 34502197

[16] Schmidt AD , de Guzman Strong C . Current understanding of epigenetics in atopic dermatitis. Exp Dermatol 2021; 30: 1150–1155. 10.1111/exd.14392 PMC8361700 34008901

[17] Pacella G , Capell BC . Epigenetic and metabolic interplay in cutaneous squamous cell carcinoma. Exp Dermatol 2021; 30: 1115–1125. 10.1111/exd.14354 PMC8324523 33844325

[18] Haber RN , Dib NG . Management of Darier disease: A review of the literature and update. Indian J Dermatol Venereol Leprol 2021; 87: 14–21. 10.25259/IJDVL_963_19 33580925

[19] Hunt M , Wang N , Pupinyo N , Curman P , Torres M , Jebril W , et al . Dantrolene corrects cellular disease features of Darier disease and may be a novel treatment. EMBO Mol Med 2024; 16: 1986–2001. 10.1038/s44321-024-00104-3 PMC11392931 39060641

[20] Schnabel CA , Selleri L , Jacobs Y , Warnke R , Cleary ML . Expression of Pbx1b during mammalian organogenesis. Mech Dev 2001; 100: 131–135. 10.1016/s0925-4773(00)00516-5 11118899

[21] Mascharak S , desJardins-Park HE , Davitt MF , Griffin M , Borrelli MR , Moore AL , et al . Preventing *Engrailed-1* activation in fibroblasts yields wound regeneration without scarring. Science 2021; 372: eaba2374. 10.1126/science.aba2374 PMC9008875 33888614

[22] Flockhart RJ , Diffey BL , Farr PM , Lloyd J , Reynolds NJ . NFAT regulates induction of COX-2 and apoptosis of keratinocytes in response to ultraviolet radiation exposure. FASEB J 2008; 22: 4218–4227. 10.1096/fj.08-113076 PMC2671982 18708588

[23] Wong PP , Pickard A , McCance DJ . p300 alters keratinocyte cell growth and differentiation through regulation of p21(Waf1/CIP1). PLoS ONE 2010; 5: e8369. 10.1371/journal.pone.0008369 PMC2805707 20084294

[24] Bai R , Guo Y , Liu W , Song Y , Yu Z , Ma X . The Roles of WNT Signaling Pathways in Skin Development and Mechanical-Stretch-Induced Skin Regeneration. Biomolecules 2023; 13: 1702. 10.3390/biom13121702 PMC10741662 38136575

[25] D’Arcy C , Kiel C . Cell Adhesion Molecules in Normal Skin and Melanoma. Biomolecules 2021; 11: 1213. 10.3390/biom11081213 PMC8391223 34439879

[26] Yu X , Zhao H , Wang R , Chen Y , Ouyang X , Li W , et al . Cancer epigenetics: from laboratory studies and clinical trials to precision medicine. Cell Death Discov 2024; 10: 28. 10.1038/s41420-024-01803-z PMC10789753 38225241

[27] Marella S , Plikus MV , Gudjonsson JE . Skin fibroblasts in health and disease: From extracellular matrix remodeling to immune regulation. J Invest Dermatol 2026; 146: 1473–1485. 10.1016/j.jid.2025.12.002 41493297

[28] Manabe T , Park H , Minami T . Calcineurin-nuclear factor for activated T cells (NFAT) signaling in pathophysiology of wound healing. Inflamm Regen 2021; 41: 26. 10.1186/s41232-021-00176-5 PMC8371293 34407893

[29] Bai R , Guo Y , Liu W , Song Y , Yu Z , Ma X . The Roles of WNT Signaling Pathways in Skin Development and Mechanical-Stretch-Induced Skin Regeneration. Biomolecules 2023; 13: 1702. 10.3390/biom13121702 PMC10741662 38136575

[30] Widelitz RB . Wnt signaling in skin organogenesis. Organogenesis 2008; 4: 123–133. 10.4161/org.4.2.5859 PMC2634258 19279724

[31] Lim X , Tan SH , Koh WLC , Chau RMW , Yan KS , Kuo CJ , et al . Interfollicular epidermal stem cells self-renew via autocrine Wnt signaling. Science 2013; 342: 1226–1230. 10.1126/science.1239730 PMC4081860 24311688

[32] Ding X , Bloch W , Iden S , Rüegg MA , Hall MN , Leptin M , et al . mTORC1 and mTORC2 regulate skin morphogenesis and epidermal barrier formation. Nat Commun 2016; 7: 13226. 10.1038/ncomms13226 PMC5095294 27807348

[33] Jevtić M , Löwa A , Nováčková A , Kováčik A , Kaessmeyer S , Erdmann G , et al . Impact of intercellular crosstalk between epidermal keratinocytes and dermal fibroblasts on skin homeostasis. Biochim Biophys Acta Mol Cell Res 2020; 1867: 118722. 10.1016/j.bbamcr.2020.118722 32302667

