# Supplementary material for: Epigenomic Characterization of Fibroblasts in Darier Disease Identifies Cross-cell-type Regulatory Gene Signatures
Source: Acta Derm Venereol. 2026 Oct 28;106:0626. doi: 10.2340/actadv.v106.adv-2026-0626 (PMC13714876; doi:10.2340/actadv.v106.adv-2026-0626)
Supplement: Supplementary file 1 [file ActaDv-106-0626-s0001.pdf]

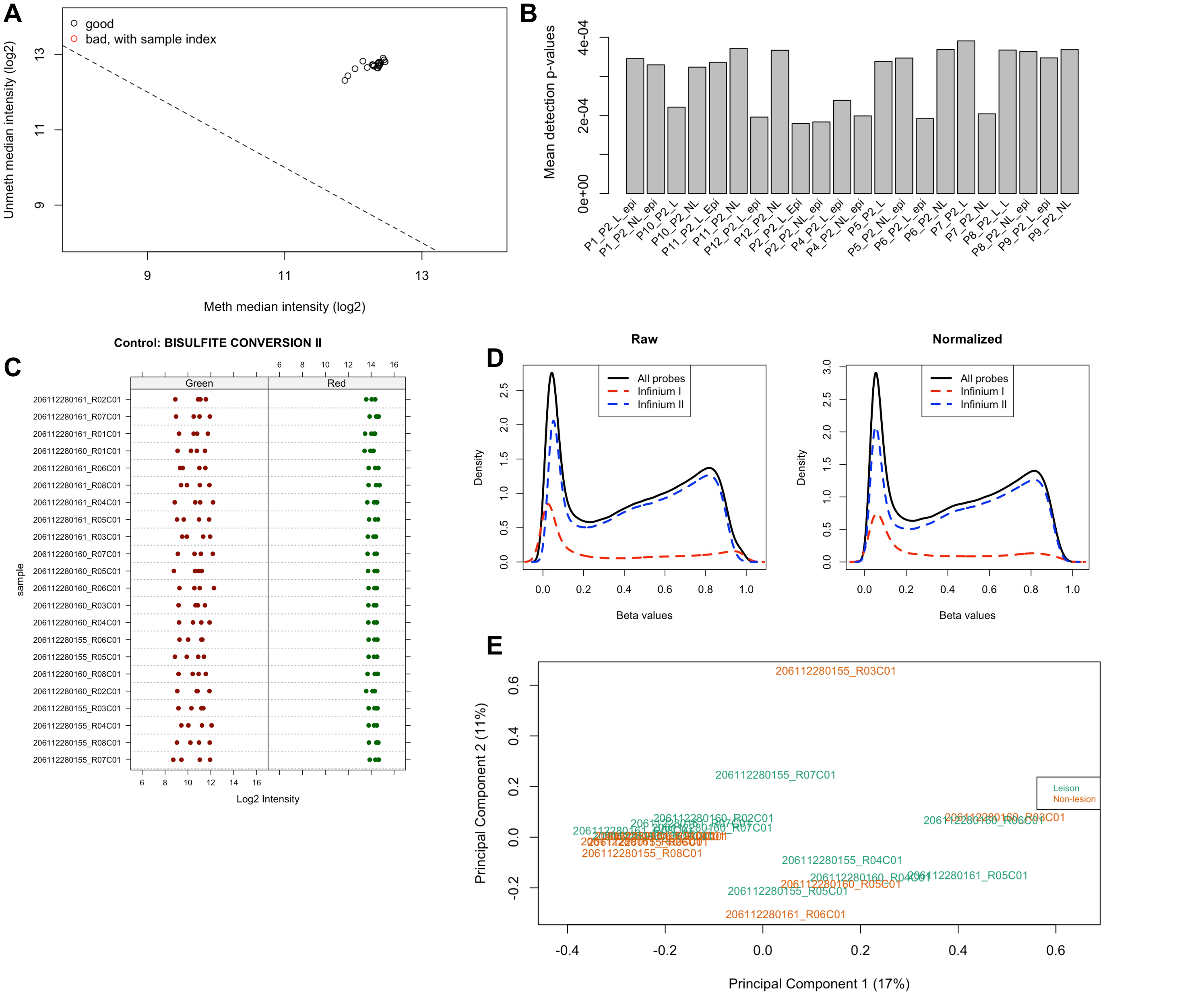

Supplementary Figure 1. Quality control of DNA methylation array data. (**A**) Scatter plot of median methylated (Meth) versus unmethylated (Unmeth) signal intensities (log2-transformed) for each sample. Each point represents one sample. Samples clustering at high intensity values indicate good hybridization quality and balanced signal intensities. (**B**) Mean detection P-values for each sample across all probes. Low mean detection P-values indicate high signal-to-noise ratios and overall good probe performance. (**C**) Built-in Illumina bisulfite conversion control probes for green and red channels, demonstrating efficient and consistent bisulfite conversion across all samples. (**D**) Density plots of β value distributions before normalization (Raw), showing characteristic differences between Infinium I and Infinium II probes. (**E**) Density plots of β value distributions after normalization, demonstrating successful correction of probe-type bias and improved alignment between Infinium I and II probes. (**F**) Principal component analysis (PCA) based on normalized β values. Samples are colored according to lesion and non-lesion groups, illustrating the absence of major outliers or batch effects across the dataset.

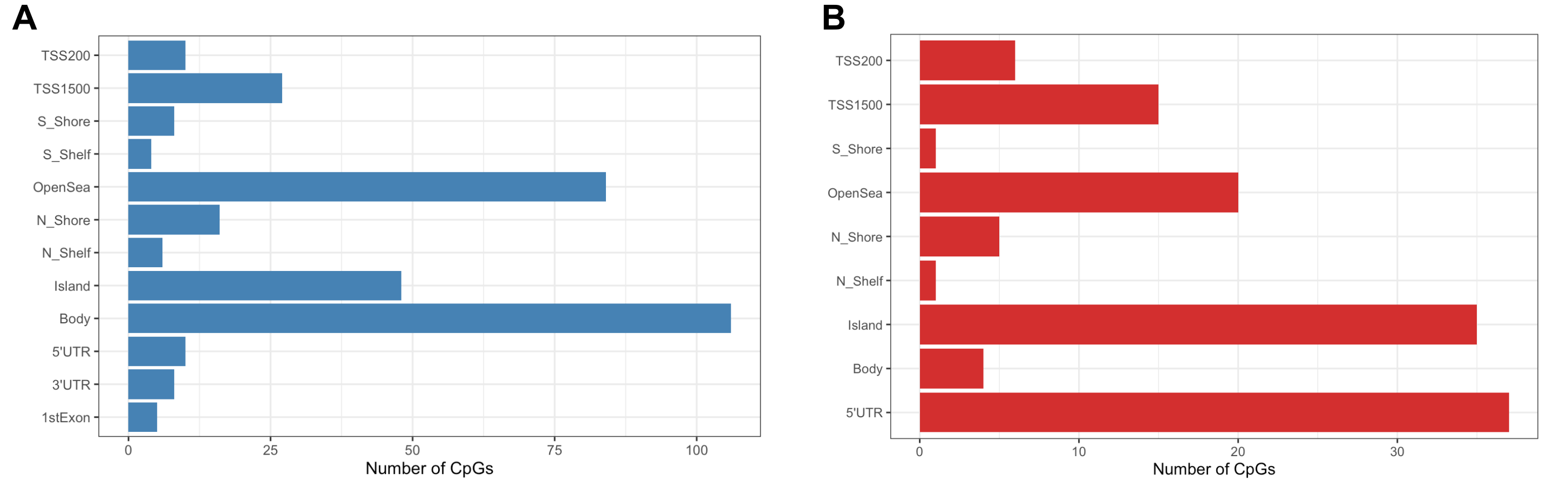

Supplementary Figure S2. Genomic distribution of CpG sites across annotated regions in STRING hub genes. Bar charts showing the number of CpG sites mapping to each genomic annotation for (**A**) hypomethylated and (**B**) hypermethylated hub genes identified from STRING protein–protein interaction network analysis.

Gene	Sequence
CTNNA1 F	TCCGATCCTCTATACTGCATCC
CTNNA1 R	TGCTGTGAGGCATCGTCTG
CREBBP F	CCTGCCACGTCACAGACTG
CREBBP R	GGCCAGAGTTACTATTGAGGAGG
PBX1 F	GACAACTCAGTGGAGCATTCA
PBX1 R	CTCTCGCAGGAGATTCATCAC
MEF2C F	GAACGTAACAGACAGGTGACAT
MEF2C R	CGGCTCGTTGTACTCCGTG
AXIN1 F	TGGAGCCCTGTGACTCGAA
AXIN1 R	GGGACACGATGCCATTGTTATC
ABL1 F	CATCACGCCAGTCAACAGTCT
ABL1 R	GTACACCCTCCCTTCGTATCT
RPTOR F	GCTAGGTGCATTGACATACAACA
RPTOR R	AGTGCTAGTTCACAGATAATGGC
PAX6 F	AACGATAACATACCAAGCGTGT
PAX6 R	GGTCTGCCCGTTCAACATC
TP73 F	GACGAGGACACGTACTACCTT
TP73 R	CTGCCGATAGGAGTCCACCA
CADM2 F	AAGAGGATGCAAATCGCAAGA
CADM2 R	CACTCCATCATCACTCCGGT
EN1 F	GTGGTCAAAACTGACTCGCAGC
EN1 R	CCGCTTGTCCTCCTTCTCGTTC
NFATC2 F	TTCCCCTGTGGCTTTTGTGT
NFATC2 R	CTGCTGCGCTAAACGTCATC

Supplementary Table 1. qPCR primer sequences used in this study. Table summarizing the primer pairs used for quantitative PCR (qPCR) analysis. For each gene, forward (F) and reverse (R) primer sequences (5'→3') are listed.
