# Supplementary material for: Epigenomic Characterization of Fibroblasts in Darier Disease Identifies Cross-cell-type Regulatory Gene Signatures
Source: Acta Derm Venereol. 2026 Oct 28;106:0626. doi: 10.2340/actadv.v106.adv-2026-0626 (PMC13714876; doi:10.2340/actadv.v106.adv-2026-0626)
Supplement: Supplementary file 2 [file ActaDv-106-0626-s0002.pdf]

Hypomethylated top genes

Gene	Function	Hypomethylation	Disorder
**EN1**	Developmental transcription factor, cell fate determination	Reactivation of developmental programs	Aberrant tissue remodeling
**HOPX**	Stem cell state maintenance	Reduced terminal differentiation	Chronic lesions, delayed resolution
**AXIN1**	Wnt/β-catenin pathway regulation	Dysregulated cell fate signaling	Abnormal epithelial differentiation
**PTP4A2**	Cell migration and proliferation	Enhanced cellular motility	Excessive tissue remodeling
**SASH1**	Signal integration and inflammatory regulation	Amplified inflammatory signaling	Chronic inflammation
**ENO1**	Glycolysis and metabolic regulation	Metabolic reprogramming	Hypermetabolic or stress-adapted states
**TAP2**	Antigen processing and MHC class I presentation	Enhanced immune activation	Autoimmune or inflammatory tendencies
**CACNB2**	Calcium channel regulation	Increased cellular excitability	Sensory or neuromuscular abnormalities
**SDK2**	Cell adhesion and signaling	Increased intercellular communication	Tissue reorganization
**NRN1**	Neuronal plasticity	Enhanced neurogenic signaling	Sensory hypersensitivity

Supplementary Table 1. Functional annotation of the top 10 hypomethylated genes in lesional DDF. Top 10 most significantly hypomethylated genes identified in lesional versus non-lesional DDF, ranked by methylation significance.

Hypermethylated top genes

Gene	Function	Hypermethylation	Disorder
**PIK3C2G**	PI3K signaling, cellular metabolism, membrane trafficking	Suppression of PI3K–Akt–mTOR signaling	Impaired tissue repair, metabolic dysregulation
**RPTOR**	Core component of mTORC1, regulates protein synthesis and autophagy	Reduced mTOR activity	Chronic tissue dysfunction, impaired regeneration
**NGFR**	Neurotrophin receptor, neuro–skin communication	Disrupted neurotrophic signaling	Sensory abnormalities, chronic pain, pruritus
**BOC**	Hedgehog signaling co-receptor, developmental regulation	Attenuated developmental signaling	Abnormal tissue patterning, hair follicle defects
**CDH23**	Cell–cell adhesion (cadherin family)	Reduced epithelial cohesion and barrier integrity	Barrier dysfunction, increased inflammation susceptibility
**FRY**	Cell polarity and cytoskeletal organization	Loss of structural stability	Tissue fragility, impaired mechanical resilience
**MYOM2**	Cytoskeletal and contractile support	Altered cellular mechanical properties	Structural instability
**SNTG2**	Cytoskeleton–signal coupling	Impaired signal transmission	Altered stress or sensory responses
**SFSWAP**	RNA splicing regulation	Dysregulated post-transcriptional control	Reduced stress adaptability
**GAK**	Endocytosis and vesicle trafficking	Impaired receptor turnover	Delayed signal responsiveness

Supplementary Table 2. Functional annotation of the top 10 hypermethylated genes in lesional DDF. Top 10 most significantly hypermethylated genes identified in lesional versus non-lesional DDF, ranked by methylation significance.
